# Self-Reference Emerges Earlier than Emotion during an Implicit Self-Referential Emotion Processing Task: Event-Related Potential Evidence

**DOI:** 10.3389/fnhum.2017.00451

**Published:** 2017-09-08

**Authors:** Haiyan Zhou, Jialiang Guo, Xiaomeng Ma, Minghui Zhang, Liqing Liu, Lei Feng, Jie Yang, Zhijiang Wang, Gang Wang, Ning Zhong

**Affiliations:** ^1^Faculty of Information Technology, Beijing University of Technology Beijing, China; ^2^Beijing International Collaboration Base on Brain Informatics and Wisdom Services, Beijing University of Technology Beijing, China; ^3^Beijing Key Laboratory of Magnetic Resonance Imaging and Brain Informatics, Beijing University of Technology Beijing, China; ^4^State Key Laboratory of Cognitive Neuroscience and Learning & IDG/McGovern Institute for Brain Research, Beijing Normal University Beijing, China; ^5^The National Clinical Research Center for Mental Disorders, Beijing Anding Hospital, Capital Medical University Beijing, China; ^6^Beijing Key Laboratory of Mental Disorders, Beijing Anding Hospital, Capital Medical University Beijing, China; ^7^Institute of Mental Health (Sixth Hospital), Peking University Beijing, China; ^8^National Clinical Research Center for Mental Disorders, Key Laboratory of Mental Health, Ministry of Health, Peking University Beijing, China; ^9^Beijing Municipal Key Laboratory for Translational Research on Diagnosis and Treatment of Dementia Beijing, China; ^10^Beijing Advanced Innovation Center for Future Internet Technology, Beijing University of Technology Beijing, China; ^11^Department of Life Science and Informatics, Maebashi Institute of Technology Maebashi, Japan

**Keywords:** emotion, self, self-reference, other-reference, ERP

## Abstract

Self-referential emotion refers to the process of evaluating emotional stimuli with respect to the self. Processes indicative of a self-positivity bias are reflected in electroencephalogram (EEG) signals at ~400 ms when the task does not require a discrimination of self from other. However, when distinguishing between self-referential and other-referential emotions is required, previous studies have shown inconsistent temporal dynamics of EEG signals in slightly different tasks. Based on the observation of early self–other discrimination, we hypothesized that self would be rapidly activated in the early stage to modulate emotional processing in the late stage during an implicit self-referential emotion. To test this hypothesis, we employed an implicit task in which participants were asked to judge the order of Chinese characters of trait adjectives preceded by a self (“I”) or other pronoun (“He” or “She”). This study aimed to explore the difference of social-related emotional evaluation from self-reference; the other pronoun was not defined to a specific person, rather it referred to the general concept. Sixteen healthy Chinese subjects participated in the experiment. Event-related potentials (ERPs) showed that there were self-other discrimination effects in the N1 (80–110 ms) and P1 (170–200 ms) components in the anterior brain. The emotional valence was discriminated in the later component of N2 (220–250 ms). The interaction between self-reference and emotional valence occurred during the late positive potential (LPP; 400–500 ms). Moreover, there was a positive correlation between response time (RT) and N1 in the self-reference condition based on the positive-negative contrast, suggesting a modulatory effect of the self-positivity bias. The results indicate that self-reference emerges earlier than emotion and then combines with emotional processing in an implicit task. The findings extend the view that the self plays a highly integrated and modulated role in self-referential emotion processing.

## Introduction

Self-referential emotion refers to the process of evaluating emotional stimuli with respect to the self (Zinck, 2008). A self-referential task in which participants are asked to judge whether the emotional personality trait words describe themselves is widely used to investigate this issue. The medial prefrontal cortex is reportedly involved in self-representation (Macrae et al., 2004; Northoff and Bermpohl, 2004; Northoff et al., 2006; Moran et al., 2009; Rameson et al., 2010; Qin and Northoff, 2011). This self-related region cooperates with the emotional limbic and frontal-parietal systems to evaluate and modulate emotion and cognition (Han and Ma, 2014; Hu et al., 2016), suggesting a complex interaction of self, emotion and general cognition during self-referential emotion processing.

Event-related potentials (ERPs) can reveal dynamic temporal patterns and clarify how self-referential emotion is processed. Emotions are usually characterized as adaptive response patterns to the emotionally significant presence of events (Russell, 2003; Scherer, 2005), and there is early emotional discrimination in the stage of 200–300 ms (Schupp et al., 2006, 2007; Kissler et al., 2007, 2009; Citron, 2012; Citron et al., 2013; Imbir et al., 2015). Self-relevant emotional information often entails positively biased processing (Fields and Kuperberg, 2015). For example, when participants are asked to judge which emotional trait words describe the self from one’s own perspective, a friend’s perspective, or a stranger’s perspective, the N400 (200–400 ms) amplitude is reduced by positive relative to negative words, both in the self-respective and friend-perspective conditions (Zhou et al., 2013; Li et al., 2016). The results from a cross-cultural study suggested that such self-positivity biases are similar in both Eastern Asian and Western populations in the late positive potential (LPP) component (350–850 ms; Cai et al., 2016). In addition, self-referential emotion could occur earlier depending on the self-esteem level (Zhang et al., 2013; Yang et al., 2014b).

However, one of the most important roles of self is to discriminate one’s own from non-self or other-related stimuli. The widely reported self-prioritization effect in perception and memory suggests a social discrimination function of self (Macrae et al., 2004, 2017; Sui et al., 2012a,b, 2015; Schäfer et al., 2015, 2016). The components of N1 (50–150 ms), P2 (about 150–250 ms), and P300 (about 300–500 ms) have shown the advantage effect for self-relevant stimuli (Zhao et al., 2009, 2011; Fan et al., 2011; Yang et al., 2012; Chen et al., 2015; Liu et al., 2016). Interestingly, self-identification is highly sensitive to temporal processing, and there is a self-relevant degree effect where high self-relevant stimuli are preferentially processed relative to those low in self-relevance (Chen et al., 2011, 2015; Guan et al., 2014). The tasks and elicited ERP components vary among studies, but the findings consistently suggest that self- and other-relevant processes could be rapidly discriminated in the very early stage. Hence, automatic self-discrimination might modulate the time course of self-referential emotion.

In an implicit self-referential processing task, participants were instructed to silently read noun words preceded by either self-related pronoun word (“my”) or non-self-related word (article word “the”). ERP analysis showed that emotion was rapidly discriminated in an early time window of early posterior negativity (EPN, 200–300 ms), regardless of whether the preceding words were self-referential or other-referential, while emotion was modulated by self-reference in the later stage of LPP (450–600 ms; Herbert et al., 2011b). The later interaction between self-reference and emotion is consistent with findings in the self-referential emotion task without discrimination of self- and other-relevant (Zhou et al., 2013; Cai et al., 2016; Li et al., 2016) and further suggest that self-reference interacts with emotion to categorize information, but after emotional discrimination. Nevertheless, the self-discrimination effect was not reported.

In addition, the time course is more complicated when self-referential emotion needs to be distinguished from other-reference emotion. In another implicit study conducted by Herbert et al. (2011a), participants were instructed to silently read unpleasant, pleasant and neutral pronoun-noun and article—noun expressions that were related to the participants themselves, related to an unknown third person, or had no self-other reference at all (“my”, “his”, or “the”). Self-related and other-related pronoun-noun pairs were differentiated at 250–350 ms, followed by the interaction between self and emotion at 350–550 ms in the anterior brain. In the posterior brain, the conditions of self and other pronouns were differentiated from the non-self-reference condition (article words) at 200–400 ms, accompanied by emotional discrimination. In another investigation with sentence reading and scenario comprehension, self and other discriminated almost automatically in the occipital (P1, 50–100 ms) and frontal (N1, 100–150 ms; P2, 200–300 ms) regions (Fields and Kuperberg, 2012). These findings demonstrate that when it is required by task demands to discriminate self from other, self-discrimination occurs earlier than the so-called adaptive emotional response, and then the self would integrate the emotional information. That means the self-referential emotion could be highly self-specialized in temporal dynamics.

Inconsistent findings were also reported. For example, when participants were asked to read two-sentence social vignettes that were either self- or other-relevant, only a self-positivity bias effect at 300–500 ms was reported (Fields and Kuperberg, 2015). In a study with Chinese participants (Chen et al., 2014), personality trait words were implicitly preceded by self or other pronouns, and participants were asked to judge word emotional valence. There was an advanced self-positivity bias in the early time window of N2 (100–200 ms), but there were no main effects of emotion or self–other discrimination. Another study using the go/no-go paradigm reported a similar interaction between self and emotion in the component of N270 (200–400 ms; Wu et al., 2014). All these studies showed a consistent interaction between self and emotion processing, while there were no main effects of self and emotion. The absence of emotion discrimination in these studies could not be explained by the task paradigms since the tasks emphasized more on emotional processing. However, the enhanced emotional processing in these tasks might facilitate the interaction between emotional information and self-reference, decreasing the processing of self–other discrimination. There is therefore a need to clarify the time course of self and emotion processing using a more implicit self-reference emotion task paradigm.

In this study, we investigated the time course of self- and other-referential emotion using a modified implicit, self-referential task paradigm (Herbert et al., 2011a,b; Chen et al., 2014). In the task, an affective personality trait word was preceded by a pronoun word to indicate self- or other-relevance, and then the participants were asked to judge whether the following Chinese character was the first or the second character in the affective word. This task might involve less emotional or semantic activation of the trait word compared to silent reading (Herbert et al., 2011a,b) or emotion judgment (Chen et al., 2014), and would decrease the interaction between emotional information and self-reference. The processing of self-referential emotion in this task would therefore be more implicit since the judgment is unrelated to self-reference and emotion. Based on the rapid and automatic self-identification effect, we hypothesized that self-related processes would be rapidly activated in the early stage to modulate emotional processing in the late stage. Both early and late ERP components were analyzed.

## Materials and Methods

### Ethics Statement

The study was approved by the Ethics Committee of Beijing Anding Hospital, Capital Medical University, Beijing, China, compliant with the ethical standards outlined in the Declaration of Helsinki. Written informed consent was obtained from each subject prior to their participation, after the nature and possible consequences of the studies were explained.

### Participants

Sixteen healthy, right-handed subjects (eight males and eight females) participated in this study. Their ages ranged from 21 to 60 (43.19 ± 13.03). The participants were all Han Chinese and lived in mainland China. All participants reported no history of neurological or psychiatric disorders.

### Materials and Procedure

A total of 96 two-character personality trait words were selected from the Chinese Affective Words System (Wang et al., 2008), and the words are listed in Supplementary Table S1. Half of the words were positive and half were negative (valence scores of 6.55 ± 0.41 and 3.29 ± 0.37, respectively; *t*_(94)_ = −41.026, *p* < 0.00001). The differences of arousal and familiarity were not significant (for arousal, positive = 4.72 ± 0.60, negative = 4.85 ± 0.60, *t*_(94)_ = 1.062, *p* = 0.291; for familiarity, positive = 5.45 ± 0.49, negative = 5.30 ± 0.41, *t*_(94)_ = −1.540, *p* = 0.127).

The selected affective personality words were combined with the self-referential factor to produce four experimental conditions: Self Positive (SP), Self Negative (SN), Other Positive (OP), and Other Negative (ON). To balance the combination effect, the positive and negative words were randomly divided into two lists and used in two experimental procedures. In the first procedure, personality word list A was only combined with the pronoun of self, and list B was only combined with the pronoun of other. In the second procedure, the combination was switched, with list A to other pronouns and list B to self pronouns. Only one of the two procedures was used for each participant. Within lists A and B, the only significant difference was for the dimension of emotional valence, not for arousal or familiarity. There were no differences between lists A and B in the three dimensions. Detailed information for the affective personality words in lists A and B is shown in Table 1.

**Table 1:** Detailed information about the affective personality trait words used in the task.

	List A			List B			*P* value (List A vs. List B)	
	Positive	Negative	*P* value	Positive	Negative	*P* value	Positive	Negative
Valence	6.489 ± 0.461	3.221 ± 0.398	<0.001	6.613 ± 0.349	3.359 ± 0.332	<0.001	0.298	0.196
Arousal	4.849 ± 0.532	4.845 ± 0.511	0.991	4.594 ± 0.653	4.857 ± 0.689	0.182	0.146	0.955
Familiarity	5.402 ± 0.539	5.319 ± 0.442	0.561	5.489 ± 0.451	5.287 ± 0.382	0.102	0.547	0.794

*Self-/other-reference and affective words were divided between Lists A and B, which were balanced in the dimensions of valence, arousal and familiarity*.

**Figure 1:**
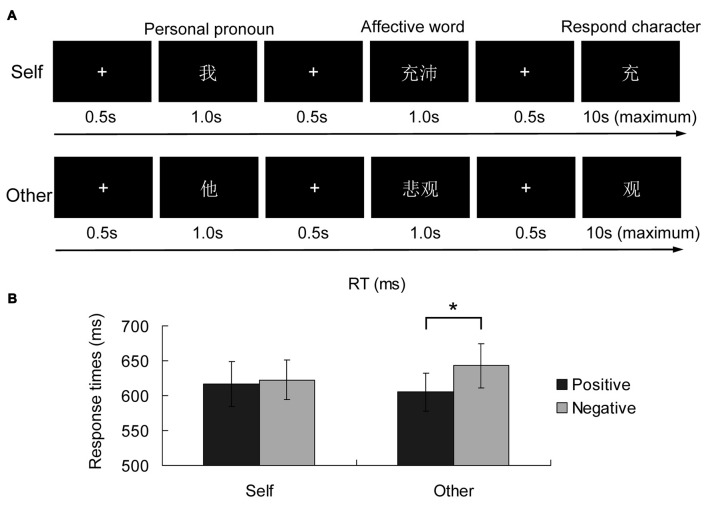
Implicit self-referential emotion task procedure **(A)** and the response times (RTs) in the experimental conditions **(B)**. After a self-referential or non-self-referential pronoun word appeared, a positive or negative personality trait word was shown on the screen, and participants were asked to judge the order of the following character in the affective word. The black and gray bars indicate the self-reference and other-reference conditions respectively. The error bars are the standard errors in each condition. *p < 0.05.

The implicit self-referential task is depicted in Figure 1A. For each trial, after a white “+” appeared in the middle of the black screen for 500 ms, a personal pronoun word appeared on the screen for 1000 ms. For the self-referential condition, it was the Chinese character of “
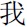
” (means I), and for the other-referential condition, it was “
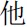
” (means He) for male participants and “
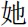
” (means She) for female participants. To compare our procedure, “the + noun” in Herbert et al.’s (2011b) study, does not make any reference to another person whereas “he/she + adjective” refers to some other person. After a “+” was shown on the screen for 500 ms, an affective personality trait word (for example, “
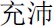
” means vigorous in the positive condition, and “
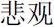
” means pessimistic in the negative condition) appeared for 1000 ms. Finally, after a “+” was on the screen for 500 ms, a Chinese character was shown for a maximum of 10,000 ms. The participants were asked to judge whether the character was the first or second character in the previous affective word, and they were instructed to press the response key with their index finger for the first character (for examples, “
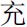
” and “
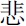
”) or their middle finger for the second character (for examples, “
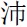
” and “
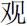
”). The character disappeared once the participant responded. The inter-stimulus interval (ISI) was 1600–2000 ms. There were a total of four blocks, each including 24 trials with an equal number of trials in the SP, SN, OP and ON conditions. The numbers of the two kinds of response types were balanced in each block. Participants practiced to familiarize themselves with the task before the formal experiment. Both the accuracy and response time (RT) were recorded during the experiment.

### Behavioral Data Analysis

First, the errors and extreme responses with RTs out of the three standard deviations (SDs) in each condition for each participant were deleted, corresponding to ~3% (41/1536) of data. Then both the accuracy and RTs in the four experimental conditions were calculated for each participant. Under each condition, the accuracy was calculated by the remaining number of data divided by the total number and RT was the average value of the remaining data. Finally, 2 (self-reference: self vs. other) × 2 (emotion: positive vs. negative) repeated measures analysis of variance (ANOVA) was performed using SPSS Statistics 20.0 (IBM, Armonk, NY, USA) to investigate the self-reference and emotional valence effects and their interaction for both accuracy and RT.

### ERP Recording and Analysis

The ERP data were recorded during the experiment in a quiet, softly lit room. Participants were instructed to sit comfortably in a seat. The distance from their eyes to the screen was about 80 cm, and the horizontal and vertical angles of view were ~5°. Brain electrical activity was recorded with a 64-electrode cap (Brain Products, Gilching, Germany) placed according to the extended International 10/20-system and referenced to the frontocentral midline electrode (FCz). The horizontal electrooculogram (HEOG) was recorded at the outer canthi, about 1.5 cm from the left eye, and the vertical electrooculogram (VEOG) was recorded about 1.5 cm below the right eye. Both the electroencephalograms (EEGs) and electrooculograms (EOGs) were collected with the electrode impedances kept below 5 kΩ. EEG and EOG signals were amplified on-line with a band-pass filtering range of 0.01–30 Hz and sampled with 1000 Hz.

The EEG signals were processed with the Brain Vision Analyzer 2.0 software package (Brain Products). All data were re-referenced to the averaged left and right mastoids (TP9 and TP10) and resampled at 250 Hz. A high-pass Butterworth filter with 0.3 Hz was applied. The EEGs were corrected for ocular artifacts using the independent component analysis (ICA) method, and both the EEG epoch for the artifacts and incorrect responses were excluded from the analysis. Event-locked ERPs were obtained by extracting an epoch beginning 200 ms before the affective words and ending 600 ms after the word’s appearance. The data were baseline corrected with respect to the mean voltage over the 200 ms preceding personality word presentation. According to the ERP waves, we did analysis separately in the anterior and posterior brain. In the anterior region, we analyzed the average amplitudes of the N1 (80–110 ms), P1 (170–200 ms), N2 (220–250 ms) and LPP (400–500 ms) components in the left (AF7, AF3, F5, F3, FC5 and FC3), middle (F2, F1, Fz, FC1, FC2 and FCz), and right (AF8, AF4, F6, F4, FC6 and FC4) areas. A 3 (location: left vs. middle vs. right) × 2 (self-reference: self vs. other) × 2 (emotion: positive vs. negative) repeated ANOVA was performed to investigate the location effect, self-reference effect, emotional valence effect and their interactions. In the posterior region, we analyzed the average amplitudes of the P1 (100–130 ms), N1 (160–190 ms), and P2 (250–280 ms) components in the left (PO7, PO3 and O1), middle (POz and Pz), and right (PO8, PO4 and O2) areas. A 3 (location: left vs. middle vs. right) × 2 (self-reference: self vs. other) × 2 (emotion: positive vs. negative) repeated measures ANOVA was performed to investigate the location effect, self-reference effect, emotional valence effect, and their interactions.

## Results

### Behavioral Results

For accuracy, there were no effects for self-reference, emotion, or the interaction between self and emotion (all *p* > 0.05). For RT, the effect of emotion was marginally significant (*F*_(1,15)_ = 3.317, *p* = 0.089), and the effect of self-reference did not reach the significance level (*p* > 0.05). However, as shown in Figure 1B, the interaction between self-reference and emotion reached the significance level (*F*_(1,15)_ = 4.931, *p* = 0.042). The simple effect analysis showed a significant emotional valence effect in the other-reference condition (*F*_(1,15)_ = 6.74, *p* = 0.020), while the emotional valence effect in the self-reference condition did not reach the significance level (*p* > 0.05).

### ERP Results

#### Results in the Anterior Brain

**Figure 2:**
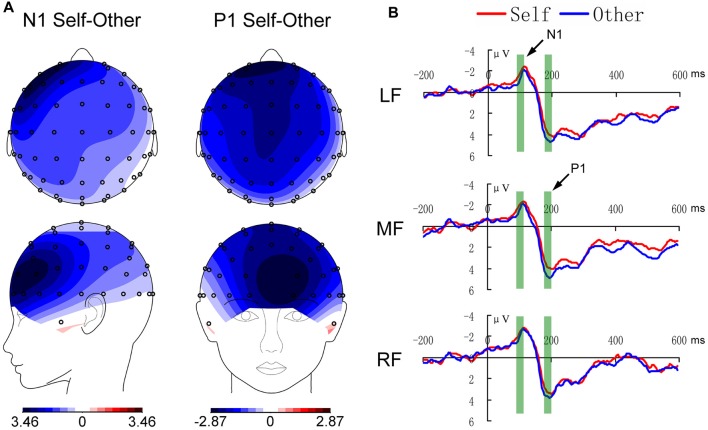
The self–other discrimination effects in the N1 (80–110 ms) and P1 (170–200 ms) components in the anterior region. **(A)** Displays the topological maps, and **(B)** shows the waveforms. LF, MF and RF indicate the left, middle and right anterior brain regions, respectively.

For the N1 component, the main effect of location was significant (*F*_(2,30)_ = 3.384, *p* = 0.047), and LSD *post hoc* test showed that the amplitude in the left region was weaker than that in the right region (*p* = 0.028). There was a self-reference effect (*F*_(1,15)_ = 6.167, *p* = 0.025), with weaker amplitude in the self-reference condition. Figure 2 shows the self-reference effect and Figure 5A shows the individual-subject effect.

**Figure 3:**
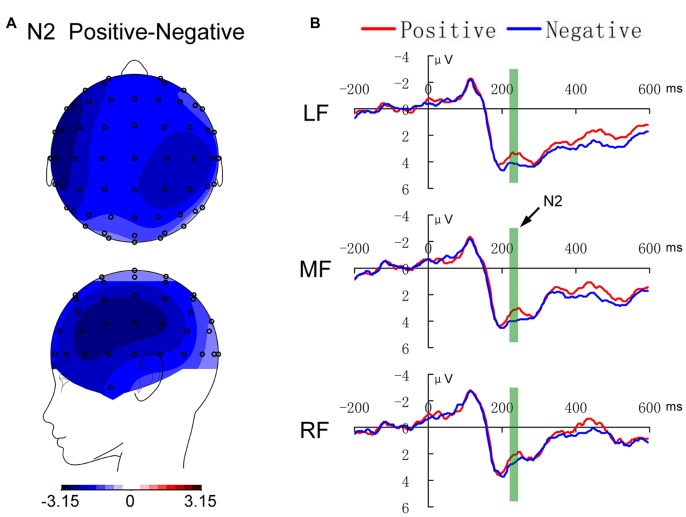
The emotional valence effect in the P2 (230–260 ms) component in the anterior brain. **(A)** Displays the topological maps, and **(B)** shows the waveforms. LF, MF and RF indicate the left, middle and right anterior brain regions, respectively.

**Figure 4:**
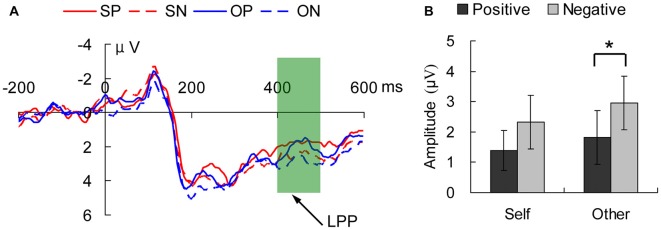
The three-way interaction in the late positive potential (LPP; 400–500 ms) component in the anterior brain. **(A)** Displays the waveforms in the four conditions and **(B)** shows the average amplitudes in the LPP time-window. The solid lines show the positive emotion and the dashed lines show the negative emotion. self positive (SP), self negative (SN), other positive (OP), other negative (ON) indicate the conditions of self-referential positive emotion, self-referential negative emotion, other-referential positive emotion and other-referential negative emotion, respectively. The error bars are the standard errors in each condition. *p < 0.05.

**Figure 5:**
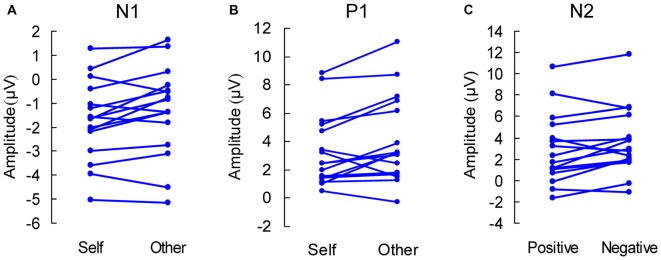
Individual-subject experimental effects in the anterior brain. **(A,B)** Display the self-reference effect in the components of N1 and P1, respectively. **(C)** Displays the emotional valence effect.

For the component of P1, there was only a self-reference effect with weaker amplitude in the self-reference condition (*F*_(1,15)_ = 5.678, *p* = 0.031), as shown in Figures 2, 5B.

For the component of N2, the effect of location was significant (*F*_(2,30)_ = 7.723, *p* = 0.002), and the LSD *post hoc* test showed that the amplitude in the right region was weaker than those in the left and middle regions (both *p* < 0.01). In addition, as shown in Figures 3, 5C, the main effect of emotion was significant (*F*_(1,15)_ = 5.560, *p* = 0.032), with weaker amplitude in the positive condition.

For the component of LPP shown in Figure 4, the effect of location reached the significance level (*F*_(2,30)_ = 23.303, *p* = 0.000), and the LSD *post hoc* test showed that the amplitude in the right region was weaker than those in the left and middle regions (both *p* < 0.01). The three-way interaction was significant (*F*_(2,30)_ = 4.399, *p* = 0.021). The simple simple effect analysis showed that in the left region, there was a marginally significant emotional valence effect in the self-reference condition (*F*_(1,15)_ = 3.54, *p* = 0.080) and a significant emotional valence effect in the other-reference condition (*F*_(1,15)_ = 4.94, *p* = 0.042); no other interactions between factors were observed (all *p* > 0.05).

#### Results in the Posterior Brain

There was no significant main effect or interaction for the P1 component in the posterior brain. For the component of N1, the only significant effect was the location (*F*_(2,30)_ = 5.624, *p* = 0.008), and the LSD *post hoc* test showed that the amplitude in the middle region was weaker than those in the left and right regions (both *p* < 0.05). For P2, there was a significant effect for location (*F*_(2,30)_ = 3.589, *p* = 0.040), and the LSD *post hoc* test showed that the amplitude in the middle region was stronger than that in the right regions (*p* = 0.002). The three-way interaction reached the significance level (*F*_(2,30)_ = 4.149, *p* = 0.026), while the simple simple effect showed no significant effects (all *p* > 0.05).

### *Post Hoc* Correlation Analysis between ERPs and RT

**Figure 6:**
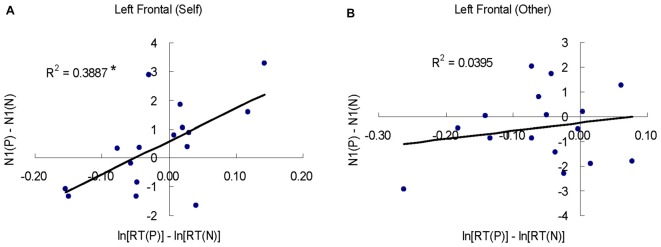
The correlations between behavioral RT and N1. **(A,B)** Indicate the correlation between RT and N1 based on the emotional valence effect in the self- and other-referential conditions. P indicates the positive condition and N is for the negative condition. *p < 0.05, Bonferroni corrected.

According to the ANOVA results, self-other was discriminated in the early components of N1 and P1; however, there was no self-positivity bias effect on behavioral performance. To explore the self-modulated emotional effect during the processing, we conducted a *post hoc* Pearson correlation analysis between ERPs (N1 and P1) and log-transformed RT data. Separate correlation analyses were performed in the self-reference and other-reference conditions in the left anterior region, where the self-other discrimination effect was strongest. To adjust for the multiple comparisons, Bonferroni correction (*n* = 4) was performed at an *α* value of 0.05. As shown in Figure 6, there was a positive correlation between RT and N1 in the self-reference condition (*r* = 0.625, *p* < 0.05, Bonferroni corrected), but no correlation in the other-reference condition (*p* > 0.05).

## Discussion

In this study, the temporal dynamics of self-referential emotion were investigated using an implicit task. The ERP patterns showed an expected and distinct self–other discrimination effect in the very early stages, with the emotional valence effect activated lightly later. Self-modulated emotion processing occurred in a later time window.

### Automatic Self–Other Identification Processing in the Early Stage

We observed a strong self–other identification effect in the early automatic processing stage for both the components of N1 (80–110 ms) and P1 (170–200 ms) in the anterior brain. This effect could not simply be attributable to the priming effect of pre-presented pronoun words. Considering the task procedure and data pattern in this study, the waveforms were slow waves ~500 ms after pronoun presentation. Before word presentation, there was an additional 500 ms of cross-presentation, which would decrease the priming effects of pronoun words to some degree. In addition, compared with results obtained using the similar “pronoun + emotional word” priming paradigms (Herbert et al., 2011b; Chen et al., 2014), neither group reported such an effect. The difference might be related to the experimental task and design. As mentioned before, enhanced emotional processing might have weakened the effect of discrimination self from other in a previous study (Chen et al., 2014); while our task paradigm is more implicit and does not emphasize self or emotional processing. In the study by Herbert et al. (2011b), the task required distinction between self- and none-self-related words (“my” vs. “the”), but there was no need to identify self-reference from other-reference. This experimental design might explain the absence of self-reference effect, because “the + noun” does not make any reference to another person. In another study conducted by the same group, where there was no priming of pronoun words but earlier self-other discrimination was observed with a contrast of self-reference and other-reference (Herbert et al., 2011a). It seems that the other-reference condition increases the social-related evaluation of emotional processing, while article words (“the”) do not have such a socially defined effect.

Early self-related identification is usually found in Chinese populations for both explicit (Sui et al., 2012c; Zhang et al., 2013) and implicit (Sui et al., 2009; Fan et al., 2011, 2013; Yang et al., 2012; Liu et al., 2016) self-referential processing. There is even a temporal sensitivity to the self-relevant degree effect in Chinese individuals (Chen et al., 2011, 2015; Guan et al., 2014). Our finding of early self-discrimination is consistent with studies of Chinese subjects (Sui et al., 2009, 2012c; Chen et al., 2011, 2015; Fan et al., 2011, 2013; Yang et al., 2012; Zhang et al., 2013; Guan et al., 2014; Liu et al., 2016) and Western populations (Herbert et al., 2011a; Sui et al., 2012c; Tacikowski et al., 2014). Research suggests that automatic processing bias towards self might not reflect stimuli familiarity but could be related to perceptual salient processing with social self-relevance, termed the self-prioritization effect (Macrae et al., 2004, 2017; Sui et al., 2012a,b, 2015; Humphreys and Sui, 2015; Schäfer et al., 2015, 2016). The self could be a center to integrate different information types at various processing stages (Sui and Humphrey, 2015), and the self-modulation effect could happen automatically or intentionally (Humphreys and Sui, 2015). Our results illustrate that the self can be rapidly identified from others to further integrate processing in a relatively automatic way.

### Emotion and Self-Modulated Emotional Processing

Although emotional processing was not emphasized in this task, there were strong emotional valence effects in the ERP data. The early component of N2 was sensitive to emotion information, with weaker amplitude in the positive condition than in the negative condition. These results are consistent with findings using an implicit task paradigm (Herbert et al., 2011a). As previously mentioned, emotional discrimination at the stage of EPN is usually regarded as an automatic adaptive response according to the degree of arousal (Schupp et al., 2006, 2007; Kissler et al., 2007, 2009; Citron, 2012; Citron et al., 2013; Imbir et al., 2015). However, there is a difference between our findings and previous results. The early emotional valence effect was observed in the anterior frontal brain, especially in the left hemisphere, while the EPN was reported in the posterior occipital brain. Actually, there is another possible reason for the difference between negative and positive stimuli. Fields and Kuperberg (2012) observed a stronger activity in the negative condition during the time window of 500–800 ms. They argued that this kind of emotional discrimination might be related to the negative bias (Taylor, 1991; Ito et al., 1998; Baumeister et al., 2001; Rozin and Royzman, 2001; Holt et al., 2009), which was related to the frontal region, whereas the arousal effect was to the posterior brain. More investigations with specified experimental designs are needed to clarify the debate surrounding the arousal and negative bias hypothesis. However, the early occurring emotional valence effect observed here suggests that there might be a strong social affective evaluation in Chinese subjects.

There was an interaction between self-reference and emotion observed in both the behavioral RT and late LPP in the left anterior brain. The results are generally consistent with the findings in previous studies using an implicit paradigm (Herbert et al., 2011a,b; Chen et al., 2014; Wu et al., 2014), but the time courses are later than those with the explicit paradigm (Zhang et al., 2013; Zhou et al., 2013; Yang et al., 2014a; Cai et al., 2016; Li et al., 2016). These patterns indicate that the increased specificity of the self-reference would bring forward the combination of self-reference and emotion information. Moreover, the simple effect analysis showed that there was significant emotional valence effect in the other-reference condition, but not the self-reference condition. Most previous studies showed the emotional effect in the self-related condition and suggested a self-positivity bias; however several others showed at least a tendency of larger emotional effects in the other-related or non-self-related conditions than in the self-related condition, but the tendency was not clearly reported or mentioned in those studies. For example, the numbers of correctly recalled items in Herbert and colleagues’ studies (Figure 4 in Herbert et al., 2011a), and the LPP amplitudes in the studies of Field and Kuperberg (Figure 5 in Fields and Kuperberg, 2012; Figure 2 in Fields and Kuperberg, 2015). One of the commonalities within the studies is the relative implicit task that imposes no direct processing demands on the self or emotion. It was argued that the self-positivity bias would emerge when making a judgment or behavioral response with regard to the self (Chambers and Windschitl, 2004; Alicke and Govorun, 2005), while the implicit paradigms used by others and in our studies might reduce access to important aspects of self-concept and could not elicit a self-positivity bias effect (Fields and Kuperberg, 2015).

However, the absence of self-positivity bias in behavior or the late component of LPP could not mean there is no self-modulated emotion during the entire process. A *post hoc* correlation analysis showed that the brain could modulate the behavior response in the early stage of N1 in the self-reference condition. Because longer RTs and greater N1 negative amplitude usually indicate strengthened effortful processing, the increased positive-negative difference in N1 shows a promoting effect on the self-referential behavior response. The correlation pattern actually reflects the self-positivity bias, and our findings suggest that the early ERP effects would contribute to the behavioral response.

### Limitations and Future Directions

With an implicit self-referential emotion task, different ERPs showed the temporal effects of self-reference and emotion and their interaction. There are some notable limitations that should be addressed in future research. First, the age range is unusually large, and the sample is relatively small. Considering that the task was simple and the variation in the behavioral RTs was not large, this might decrease the age effect to some degree. However, a larger and more homogenous sample would increase the power of the findings. Second, results based on the pronoun priming paradigm need more consideration. One is about the self-reference effect. As discussed above, the influence of priming paradigm was weakened to some degree, and there is considerable evidence of early self–other discrimination. However, caution is needed when considering the priming effect. Another issue is the other-reference condition. It seems that the other-reference increased the social-related evaluation of emotional processing (Herbert et al., 2011a), while article words (“the”) did not have such a socially defined effect (Herbert et al., 2011b). Our study mainly focused on the temporal dynamics on the self- and other-referential emotion. However, the other pronoun was not defined to a specific person such as a friend, a stranger or mother, which would affect the processing for self-referential emotion (Zhou et al., 2013; Li et al., 2016). Finally, some results seem to be related to specific culture-related features. For example, both self–other discrimination and emotional valence effects emerged earlier than in Western subjects. The Eastern Asian cultures foster interdependent self-construal, relying on how others perceive and evaluate the self (Ma et al., 2014), so it is more influenced by social context information (Kitayama and Uskul, 2011; de Greck et al., 2012; Sui et al., 2012c; Han and Ma, 2014, 2015; Ma et al., 2014; Park and Kitayama, 2014). The context-inference processing strategy would increase highly sensitive discrimination between self and others in Chinese individuals. Furthermore, Chinese subjects usually show higher sensitivity to public or social evaluation and are more anxious (Liew et al., 2011), which would increase their sensitivity to emotional processing. However, the general framework is emphasized in social neuroscience (Gaertner et al., 2012), such as with the hypothesis of interdependent vs. dependent (Markus and Kitayama, 1991). As mentioned before, similar self-referential processing was observed in both Eastern and Western populations (Fields and Kuperberg, 2012; Herbert et al., 2013; Schindler et al., 2014; Tacikowski et al., 2014; Cai et al., 2016). Further studies with cross-cultural paradigms are needed to examine temporal patterns during self-referential emotion processing, which would be helpful to further clarify the roles of related factors.

## Conclusion

An implicit self-referential emotion task was used to investigate the time-course of self and emotion processing in Chinese subjects. ERPs showed that self-reference effect occurred in the N1 and P1 components in the anterior brain, earlier than the emotional valence effect in the component of N2. Their interaction was in the LPP component. A correlation pattern was observed between N1 and RT. The findings suggest that self-modulated emotional processing occurs in a rapid and automatic way in the Chinese population.

## Author Contributions

HZ, JG, LF, GW and NZ: conceived and designed the experiments. HZ, JG, XM, MZ, LL, LF and JY: performed the experiments. HZ, JG and ZW: analyzed the data. HZ, JG and NZ: wrote the article.

## Conflict of Interest Statement

The authors declare that the research was conducted in the absence of any commercial or financial relationships that could be construed as a potential conflict of interest.
