# Supplementary material for: Self-Reference Emerges Earlier than Emotion during an Implicit Self-Referential Emotion Processing Task: Event-Related Potential Evidence
Source: Front Hum Neurosci. 2017 Sep 8;11:451. doi: 10.3389/fnhum.2017.00451 (PMC5596083; doi:10.3389/fnhum.2017.00451)
Supplement: Supplementary file 1 [file Data_Sheet_1.docx]

**Appendix**

**Table** Self-referential emotion task materials. We selected 96 two-character personality trait words from the Chinese Affective Words System (Wang, Zhou, & Luo, 2008). Half of the words were positive and half were negative, and the meanings of the words in English are listed in the third column. The scores of arousal and familiarity were matched.

| Order | Positive | Meaning | Valence | Arousal | Familiarity |  | Negative | Meaning | Valence | Arousal | Familiarity |
| --- | --- | --- | --- | --- | --- | --- | --- | --- | --- | --- | --- |
| 1 | 资深 | senior | 5.83 | 4.81 | 4.67 |  | 自私 | selfish | 2.71 | 4.73 | 5.26 |
| 2 | 冷静 | calm | 5.84 | 3.80 | 5.76 |  | 虚伪 | hypocritical | 2.73 | 5.10 | 5.09 |
| 3 | 威武 | mighty | 5.87 | 5.12 | 4.78 |  | 烦恼 | annoyed | 2.73 | 4.92 | 5.96 |
| 4 | 严谨 | rigorous | 5.88 | 4.05 | 5.06 |  | 暴躁 | petulant | 2.85 | 5.76 | 5.31 |
| 5 | 坚决 | determined | 5.88 | 5.02 | 5.27 |  | 悲观 | pessimistic | 2.87 | 4.89 | 5.37 |
| 6 | 知足 | satisfied | 5.96 | 4.06 | 5.40 |  | 粗俗 | crude | 2.90 | 4.63 | 4.63 |
| 7 | 果敢 | decisive | 5.98 | 4.75 | 4.83 |  | 消沉 | depressed | 2.93 | 4.80 | 5.34 |
| 8 | 超脱 | detached | 6.04 | 4.83 | 4.58 |  | 混乱 | confused | 3.01 | 5.40 | 4.99 |
| 9 | 显赫 | illustrious | 6.08 | 5.17 | 4.69 |  | 危险 | risky | 3.02 | 6.96 | 5.43 |
| 10 | 称职 | competent | 6.10 | 4.23 | 5.18 |  | 懒惰 | slothful | 3.03 | 4.34 | 5.71 |
| 11 | 无畏 | fearless | 6.12 | 5.36 | 4.88 |  | 哀愁 | sad | 3.03 | 5.08 | 5.11 |
| 12 | 细致 | meticulous | 6.16 | 4.22 | 5.45 |  | 郁闷 | gloomy | 3.03 | 4.95 | 6.23 |
| 13 | 茁壮 | sturdy | 6.31 | 4.10 | 4.81 |  | 荒谬 | ridiculous | 3.05 | 5.04 | 4.62 |
| 14 | 好奇 | curious | 6.32 | 5.15 | 6.10 |  | 困倦 | sleepy | 3.05 | 3.83 | 5.17 |
| 15 | 充沛 | vigorous | 6.41 | 4.43 | 5.35 |  | 沮丧 | frustrated | 3.06 | 4.89 | 5.27 |
| 16 | 非凡 | extraordinary | 6.46 | 5.57 | 5.15 |  | 无情 | ruthless | 3.06 | 5.11 | 5.01 |
| 17 | 从容 | deliberate | 6.46 | 3.76 | 5.25 |  | 恼火 | annoyed | 3.06 | 6.11 | 5.47 |
| 18 | 稳重 | stable | 6.47 | 3.51 | 5.49 |  | 孤单 | lonely | 3.11 | 4.14 | 5.69 |
| 19 | 高效 | efficient | 6.48 | 5.27 | 5.89 |  | 窘迫 | embarrassed | 3.12 | 4.86 | 4.58 |
| 20 | 精干 | skilled | 6.49 | 4.97 | 5.02 |  | 落后 | outdated | 3.13 | 4.83 | 5.56 |
| 21 | 奔放 | liberated | 6.51 | 5.33 | 4.98 |  | 自卑 | Self-abased | 3.13 | 4.75 | 5.55 |
| 22 | 安详 | peaceful | 6.53 | 3.55 | 5.25 |  | 惆怅 | melancholy | 3.13 | 4.71 | 4.74 |
| 23 | 朴实 | modest | 6.53 | 3.98 | 5.64 |  | 苛刻 | harsh | 3.15 | 5.30 | 4.46 |
| 24 | 无私 | selfless | 6.57 | 4.76 | 5.65 |  | 失意 | frustrated | 3.18 | 4.68 | 5.43 |
| 25 | 端庄 | elegant | 6.60 | 4.13 | 5.31 |  | 穷苦 | impoverished | 3.26 | 4.91 | 5.21 |
| 26 | 用功 | diligent | 6.60 | 4.72 | 6.02 |  | 草率 | hasty | 3.28 | 4.91 | 5.43 |
| 27 | 雄厚 | abundant | 6.62 | 4.80 | 4.75 |  | 衰弱 | feeble | 3.29 | 4.07 | 4.69 |
| 28 | 宽厚 | charitable | 6.65 | 4.27 | 5.47 |  | 枯燥 | boring | 3.31 | 4.10 | 5.50 |
| 29 | 坦诚 | straight-out | 6.69 | 4.09 | 5.65 |  | 别扭 | awkward | 3.33 | 4.84 | 5.20 |
| 30 | 温和 | mild | 6.71 | 3.68 | 6.11 |  | 糊涂 | confused | 3.34 | 4.50 | 5.50 |
| 31 | 卓越 | distinguished | 6.71 | 5.06 | 5.30 |  | 不满 | dissatisfied | 3.35 | 5.32 | 6.01 |
| 32 | 舒畅 | comfortable | 6.77 | 4.41 | 5.49 |  | 难堪 | embarrassed | 3.37 | 5.38 | 5.25 |
| 33 | 爽朗 | chipper | 6.78 | 4.71 | 5.85 |  | 轻率 | rash | 3.39 | 4.52 | 5.16 |
| 34 | 正直 | righteous | 6.80 | 4.50 | 5.79 |  | 疲乏 | fatigued | 3.40 | 4.18 | 5.35 |
| 35 | 俊秀 | pretty | 6.83 | 4.68 | 5.38 |  | 呆板 | stiff | 3.48 | 3.73 | 4.79 |
| 36 | 活泼 | lively | 6.87 | 4.81 | 6.20 |  | 紧张 | nervous | 3.49 | 5.61 | 6.05 |
| 37 | 热心 | warm-hearted | 6.88 | 5.06 | 6.13 |  | 慌忙 | hurried | 3.50 | 4.76 | 5.63 |
| 38 | 杰出 | outstanding | 6.89 | 5.49 | 5.48 |  | 麻烦 | cumbersome | 3.55 | 5.31 | 6.00 |
| 39 | 勇敢 | brave | 6.90 | 5.65 | 5.79 |  | 渺小 | insignificant | 3.56 | 3.73 | 5.03 |
| 40 | 旺盛 | energetic | 6.92 | 4.77 | 5.60 |  | 坎坷 | turbulent | 3.66 | 5.23 | 5.21 |
| 41 | 浪漫 | romantic | 6.97 | 5.54 | 6.12 |  | 固执 | stubborn | 3.69 | 5.03 | 5.52 |
| 42 | 高雅 | elegant | 7.01 | 5.35 | 5.37 |  | 曲折 | tortuous | 3.69 | 4.90 | 5.27 |
| 43 | 机智 | intelligent | 7.08 | 5.59 | 5.82 |  | 茫然 | blank | 3.74 | 3.92 | 5.01 |
| 44 | 优雅 | graceful | 7.09 | 4.52 | 5.28 |  | 散漫 | desultory | 3.75 | 4.27 | 4.80 |
| 45 | 聪明 | clever | 7.10 | 5.11 | 6.63 |  | 可怜 | poor | 3.91 | 4.82 | 5.55 |
| 46 | 渊博 | profound | 7.15 | 5.60 | 4.85 |  | 惭愧 | ashamed | 3.93 | 4.72 | 5.77 |
| 47 | 高尚 | noble | 7.19 | 5.66 | 5.39 |  | 艰辛 | laborious | 4.15 | 4.91 | 5.09 |
| 48 | 乐观 | optimistic | 7.37 | 4.62 | 6.52 |  | 急切 | impatient | 4.42 | 5.40 | 5.59 |
